# Supplementary material for: Interactive Effect of Biological Agents Chitosan, Lentinan and Ningnanmycin on Papaya Ringspot Virus Resistance in Papaya (Carica papaya L.)
Source: Molecules. 2022 Nov 2;27(21):7474. doi: 10.3390/molecules27217474 (PMC9656423; doi:10.3390/molecules27217474)
Supplement: Supplementary file 1 [file molecules-27-07474-s001.zip › molecules-1997565-supplementary.pdf]

Supplementary Material

Interactive Effect of Biological Agents Chitosan, Lentinan and Ningnanmycin on Papaya Ringspot Virus Resistance in Papaya (*Carica papaya* L.)

Heling Fan [1,2], Xingxiang Yan [1], Mingqing Fu [1], Difa Liu [1,2], Abdul Waheed Awan [1,4], Ping Chen [3], Syed Majid Rasheed [4], Ling Gao [2,*] and Rongping Zhang [1,*]

[1] College of Tropical Crops, Hainan University, Haikou, Hainan Province, China
[2] Tropical Crops Genetic Resources Institute (CATAS), Danzhou 571737, China
[3] College of Horticulture, Hainan University, Haikou, Hainan Province, China
[4] Department of Agriculture, Bacha Khan University, Charsadda, Pakistan
* Correspondence: gaoling_0898@163.com (L.G.); 990837@hainanu.edu.cn (R.Z.)

Table S1. Primers for quantitative real-time PCR.

Gene ID	Sequence information
CpActin-QF	5'-AGGCAGGCAAGAGAAGAT-3'
CpActin-QR	5'-TTCATACCGAGTAGCGATTC-3'
evm.TU.supercontig_20.79-QF	5'-TCCCCCCAAGATTTCCTGA-3'
evm.TU.supercontig_20.79-QR	5'-GTTGGTTGGCGTAGTTCTGC-3'
evm.TU.supercontig_44.71-QF	5'-CTAATCGCAGAAGCCAATCAT-3'
evm.TU.supercontig_44.71QR	5'-CCTCACCCCTCTGTACGACC-3'
evm.TU.supercontig_42.87-QF	5'-ACCCGTGTCGGCTTCTACTC-3'
evm.TU.supercontig_42.87-QR	5'-CCATCACAACCACGAACAAAAC-3'
evm.TU.supercontig_77.100-QF	5'-GGTTCAAAGCTATTGCTCTTACAGT-3'
evm.TU.supercontig_77.100-QR	5'-AACCCTCAAAGTTCTTCAATGTC-3'
evm.TU.supercontig_2.212-QF	5'-GAGACCTTTGATGGATTTCGTG-3'
evm.TU.supercontig_2.212-QR	5'-GTTTTCCCACTAAGTCCCACAT-3'
evm.TU.supercontig_118.34-QF	5'-AGCATTGGACAGGCTCTTTTC-3'
evm.TU.supercontig_118.34-QR	5'-CGCTTGTTCCATCTGGTGTAAT-3'
evm.TU.supercontig_19.185-QF	5'-AGAACCTTGCGAAGAATGTAGC-3'
evm.TU.supercontig_19.185-QR	5'-CAACTCACACTCTCTGAACCTCTG-3'

Table S2. qPCR reaction system.

2 ×qPCR Master Mix	10 μL
Forward primer（10 μM）	0.3 μL
Reverse primer（10 μM）	0.3 μL
cDNA template	1.0 μL
ddH_2O	8.4 μL

Table S3. Table of RNA detection results.

Sample name	Concentration (ng μL-1)	Volume (μL)	Overall amount (μg)	OD260/280	OD260/230	28S/18S	RIN	Detection results
CK1	1346.84	50	67.34	2.024	1.948	1.2	7.1	A
CK2	1332.24	50	66.61	2.036	1.709	1.4	8,0	A
CK3	1270.48	50	63.52	2.027	2.393	1.3	7.9	A
T2_1	2155.20	50	107.76	2.019	2.436	1.1	7.1	A
T2_2	759.00	50	37.95	2.014	2.348	1.3	7.6	A
T2_3	368.28	50	18.41	1.979	1.677	0.4	7.3	A

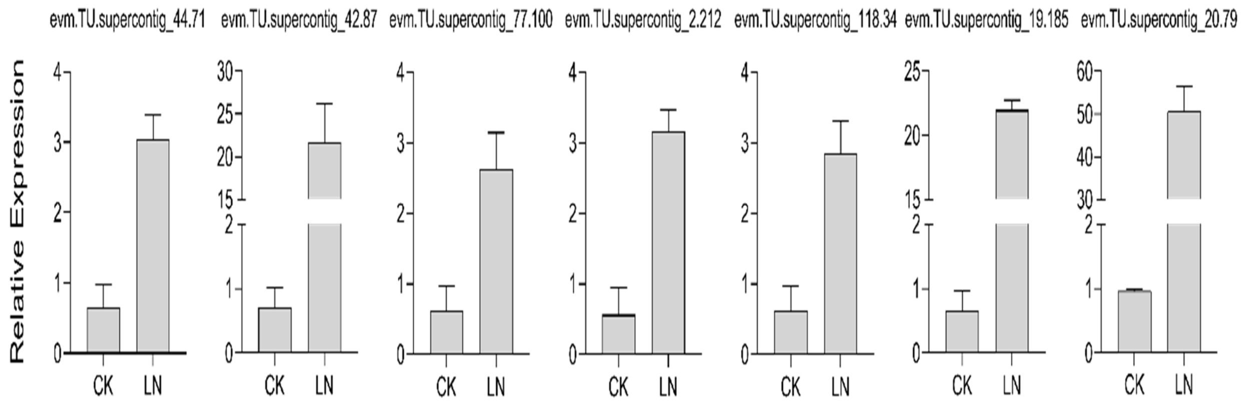

Figure S4. Verification assay of 7 differentially expressed genes (DEGs). The statistical significance of the differences was confirmed by analysis of variance (ANOVA).
